# Catastrophic adult-onset Still’s disease as a distinct life-threatening clinical subset: case–control study with dimension reduction analysis

**DOI:** 10.1186/s13075-021-02631-7

**Published:** 2021-10-11

**Authors:** Anaïs Wahbi, Benoît Tessoulin, Cédric Bretonnière, Julien Boileau, Dorothée Carpentier, Olivier Decaux, Laurence Fardet, Guillaume Geri, Pascal Godmer, Cécile Goujard, Hervé Maisonneuve, Arnaud Mari, Jacques Pouchot, Jean-Marc Ziza, Sophie Georgin-Lavialle, Mohamed Hamidou, Antoine Néel

**Affiliations:** 1 Service de Médecine Interne, PHU3, Centre Hospitalier Universitaire de Nantes — Hôtel-Dieu, 1 Place Alexis Ricordeau, 44093 Nantes, France; 2 Service d’Hématologie, PHU1, CHU Hôtel-Dieu, 44093 Nantes, France; 3 Service de Pneumologie, PHU2, CHU de Nantes, 44093 Nantes, France; 4 UPRES EA 3826, Faculté de Médecine, Université de Nantes, 44035 Nantes, France; 5 Service de Médecine, CH de Morlaix, 29672 Morlaix, France; 6 Service de Réanimation Médicale, CHU de Rouen, 76031 Rouen, France; 7 Service de Médecine Interne, CHU de Rennes, 35033 Rennes, France; 8 Service de Dermatologie, Hôpital Henri Mondor, 94000 Créteil, France; 9 Service de Réanimation Médicale, CHU Cochin, AP-HP, 75012 Paris, France; 10 CH Bretagne-Atlantique, 56000 Vannes, France; 11 Service de Médecine Interne, CHU Bicêtre, AP-HP, 94270 Kremlin-Bicêtre, France; 12 Service de Médecine Interne, CHD Vendée, 85925 La Roche-sur-Yon, France; 13 Service de Réanimation, Hôpital Yves Le Foll, 22000 St Brieuc, France; 14 Service de Médecine Interne, Hôpital Européen Georges Pompidou, AP-HP, 75908 Paris, France; 15 Service de Médecine Interne-Rhumatologie, Groupe Hospitalier Diaconesses-Croix-Saint-Simon, 75020 Paris, France; 16 Service de Médecine Interne, CHU Tenon, AP-HP, 75020 Paris, France

**Keywords:** Adult-onset Still’s disease, Reactive haemophagocytic syndrome, Shock, Acute respiratory failure, Differential diagnosis, Intensive care unit, Case–control study, Clusters, Cytokine storm

## Abstract

**Objectives:**

Adult-onset Still’s disease (AOSD) is a rare systemic inflammatory disorder. Diagnosing AOSD can be challenging, as disease presentation and clinical course are highly heterogeneous. For unclear reasons, a few patients develop life-threatening complications. Our objective was to determine whether these cases resulted from therapeutic delay or could represent a peculiar AOSD subset.

**Methods:**

We conducted a multicentre retrospective study of 20 AOSD patients with organ failure requiring intensive care unit admission and 41 control AOSD patients without organ failure. Clinico-biological data at hospital admission were explored using supervised analyses and unsupervised dimension reduction analysis (factor analysis of mixed data, FAMD).

**Results:**

Disease duration before admission was shorter in patients with life-threatening AOSD (median, 10 vs 20 days, *p* = 0.007). Disease duration before AOSD therapy initiation also tended to be shorter (median, 24 vs 32 days, *p* = 0.068). Despite this shorter disease duration, FAMD, hierarchical clustering and univariate analyses showed that these patients exhibited distinctive characteristics at first presentation, including younger age; higher frequency of splenomegaly, liver, cardiac and/or lung involvement; less frequent arthralgia; and higher ferritin level. In multivariate analysis, 3 parameters predicted life-threatening complications: lack of arthralgia, younger age and shorter time between fever onset and hospitalisation.

**Conclusion:**

This study suggests that life-threatening complications of AOSD occur very early, in a peculiar subset, which we propose to name catastrophic adult-onset Still’s disease (CAOSD). Its exact burden may be underestimated and remains to be clarified through large multicentre cohorts. Further studies are needed to identify red flags and define the optimal therapeutic strategy.

## Introduction

Adult-onset Still’s disease (AOSD) is a rare systemic inflammatory disorder of unknown aetiology. AOSD typically affects previously healthy young adults and presents with high-grade fever, evanescent rash, sore throat, arthromyalgia, arthritis, serositis, discrete lymphadenopathy, hepato-splenomegaly, neutrophilic leukocytosis, hepatic cytolysis and high serum ferritin [1].

There is no single biological or pathological finding specific for AOSD. Thus, differential diagnosis can be broad (infection, malignancy, autoimmunity, etc.), depending on the patient’s presentation. AOSD is an experience-based diagnosis but several useful diagnostic criteria have been reported [2–4]. The modified Yamaguchi and Fautrel criteria provide high sensitivity and specificity [4]. Most studies on AOSD prognosis have focused on the long-term disease course, which can be monocyclic, polycyclic and/or complicated by a chronic erosive polyarthritis [5–8]. A few patients develop organ complications that can be life-threatening, but little is known about this subgroup of patients. In Europe and North America, the overall mortality rate is classically 0 to 3%, but two recent retrospective series from Korea and Italy reported a concerning disease-related mortality in AOSD (18.7% and 16%, respectively) [9, 10].

We recently reported the analysis of a multicentre series of patients requiring intensive care unit (ICU) admission due to AOSD-related organ failure [11]. Strikingly, these complications mostly occurred during the first AOSD flare, in patients admitted to hospital because of fever of unknown origin, who developed secondary histiocytic lympho-histiocytosis (sHLH), shock, cardiac failure, respiratory distress, coagulopathy, severe hepatitis, or even multiorgan failure (MOF) before the diagnosis of AOSD was eventually made. Thus, we propose to refer to these cases as catastrophic adult-onset Still disease (CAOSD). Most cases exhibited significant diagnostic and therapeutic inertia, and 2 had a fatal outcome.

The objectives of the present study were, firstly, to assess whether CAOSD is the consequence of therapeutic delay or could represent a peculiar disease subset and, secondly, to identify simple red flags, i.e. characteristics that could indicate a risk of life-threatening complications in AOSD.

## Patients and methods

### Study population and data collection

The CAOSD group included 20 patients from a recently reported multicentre case series [11]. These patients were admitted to an ICU between 1997 and 2014 due to AOSD. Inclusion criteria were (i) admission to ICU due to AOSD-related organ failure; (ii) AOSD diagnosis fulfilling the Yamaguchi [2] and/or Fautrel [3] criteria; (iii) exclusion of differential diagnoses, including infection, malignancy and other systemic immune-mediated disorders; (iv) age at AOSD diagnosis > 18 years; and (v) organ failure requiring organ supporting therapeutic intervention, including vasopressor agents, pericardial drainage, mechanical ventilation, renal replacement therapy (RRT) or plasmatherapy. Exclusion criteria were ICU admission without organ failure or for reasons other than AOSD. Key features of CAOSD are reported in Table 1. A control group was recruited, comprising 41 AOSD patients admitted to Nantes University Hospital during a similar period (2000–2015) for a first AOSD flare. Inclusion criteria were (i) AOSD diagnosis fulfilling the Yamaguchi [2] and/or Fautrel [3] criteria; (ii) exclusion of differential diagnoses, including infection, malignancy and other systemic immune-mediated disorders; (iii) age at AOSD diagnosis > 18 years; and (iv) absence of organ failure. For both groups, initial clinical data were extracted from charts and collected using a standardised form by one of the investigators (AW). This observational study was performed in accordance with the Declaration of Helsinki, European Union and French ethical regulations (reference methodology MR003: retrospective study of anonymised data with ethics approval waiver).

**Table 1:** Key features of catastrophic adult-onset Still disease

Adult-onset Still disease	
Yamaguchi and/or Fautrel criteria	
Usually new-onset disease	
AND ≥ 1 organ failure requiring ICU management	
Acute respiratory failure, caused by	
Lung infiltrate	
Pleural effusion	
Cardio-circulatory failure	
Cardio-circulatory failure, caused by	
Non-cardiogenic shock	
Myocarditis	
Tamponade	
Haematologic disorder, caused by	
Disseminated intravascular coagulation	
Haemophagocytosis	
Thrombotic microangiopathy	
AND no alternative cause	
No infection	
No drug reaction	
No malignancy	

*ICU*, intensive care unit

### Statistical analyses

Time from first symptoms to hospital admission, time from admission to AOSD treatment initiation (i.e. NSAID or corticosteroids) and time from first symptoms to AOSD treatment initiation were compared between the 2 groups using the Mann–Whitney test. Survival curves were obtained using the Kaplan–Meier method (GraphPad Prism).

A factor analysis for mixed data (FAMD) was performed on patients’ initial data. FAMD is a principal component method which takes into account both qualitative and quantitative variables [12]. This algorithm of reduction of dimensionality sums up major variance/inertia of the variables and projects it on low-dimension planes, thus permitting easy visual interpretation of the data in a concise fashion. It also reduces the background noise of variables and permits downstream analysis, such as clustering. When missing data occurred, they were imputed by principal component analysis and multiple component analysis for quantitative and qualitative variables, respectively, in order to reduce interpolation bias [13]. Severity status was not included in axis construction. Hierarchical cluster analysis was performed on the FAMD coordinates on the first 5 dimensions, using an ascendant algorithm on the Euclidean distances between points and a Ward link. The optimal number of clusters was determined by the elbow method of the within-cluster sum of square with ascendant hierarchical clustering. Best number of clusters was further automatically confirmed by majority vote among 23 indices (NbClust package). All statistical analyses were performed within R-3.4.9 environment, using the FactoMineR, factoextra and missMDA packages [14, 15].

Univariate analyses were performed using the Mann–Whitney test and Fisher test, for quantitative and qualitative variables, respectively. A multiple logistic regression model was built, using variables with less than 10% of missing data. If variables with missing data were to be included, the prevalence of missing values had to be equally balanced between CAOSD and control cases. A backwise selection of variables was performed. Choice of the best multivariate model was based on maximisation of the Bayesian criterion information (BIC). Statistical testing was bilateral and was considered significant below 0.05.

## Results

### Catastrophic adult-onset Still disease is not due to therapeutic delay

**Fig. 1:**
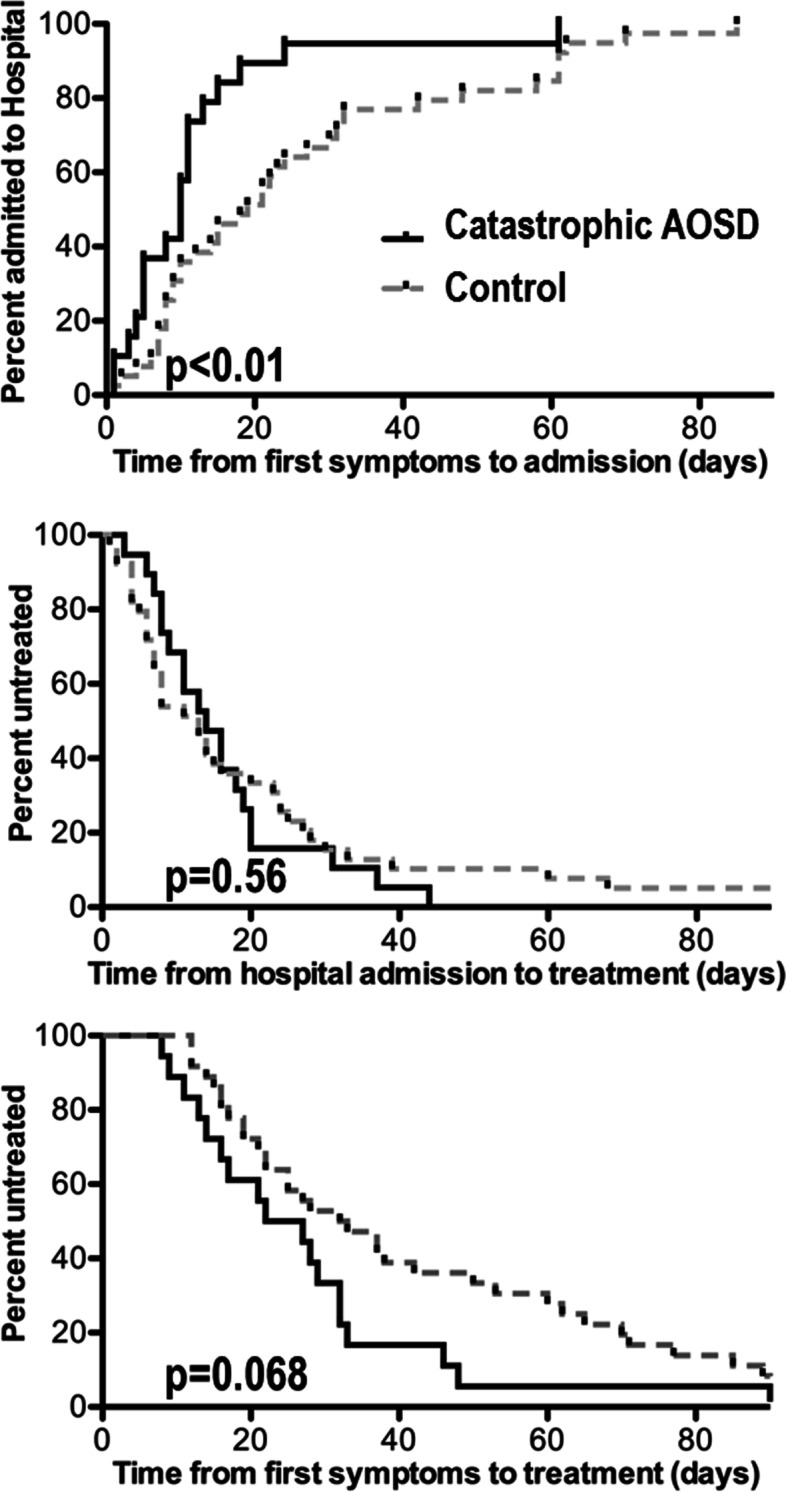
Timing of hospital admission and treatment initiation in catastrophic (black curve) versus uncomplicated (grey dotted curve) AOSD. Disease duration prior to hospital admission was significantly shorter in the catastrophic AOSD group (upper panel). Delay between hospital admission and initiation of AOSD therapy was similar in both groups (middle panel). Disease duration prior to initiation of AOSD therapy tended to be shorter in catastrophic AOSD patients (lower panel)

We first hypothesised that life-threatening complications could occur in AOSD patients that experienced unusual therapeutic delay. First-line therapy was corticosteroids in all CAOSD cases. NSAID was only used in control cases (*n* = 8, of whom 6 eventually required corticosteroids). The timing of hospital admission and that of AOSD treatment initiation are shown in Fig. 1. We found that disease duration prior to initiation of therapy tended to be shorter in the CAOSD group when compared to the control group (median, 24 [*IQR*, 14–32] days vs 32 [*IQR*, 19–64], *p* = 0.068). The delay between hospital admission and initiation of therapy in the CAOSD group was similar to that in the control group (median, 14 days [*IQR*, 8–20] vs 13 [*IQR*, 6–25], *p* = 0.56), suggesting that life-threatening complications were not due to a lengthier diagnostic work-up. Interestingly, disease duration prior to hospital admission was significantly shorter in the CAOSD group (10 days [*IQR*, 5–13] vs 20 [*IQR*, 8–32], *p* = 0.007), suggesting that these patients had a more acute disease onset.

### Multi-dimensional reduction analysis suggests that CAOSD is a distinct disease subset

Having shown that CAOSD does not result from therapeutic delay, we wanted to determine whether these patients could represent a distinct subset even at the time of hospital admission. All clinical and laboratory variables recorded on admission in both groups, i.e. before patients with CAOSD had developed organ failure, were submitted to FAMD.

**Fig. 2:**
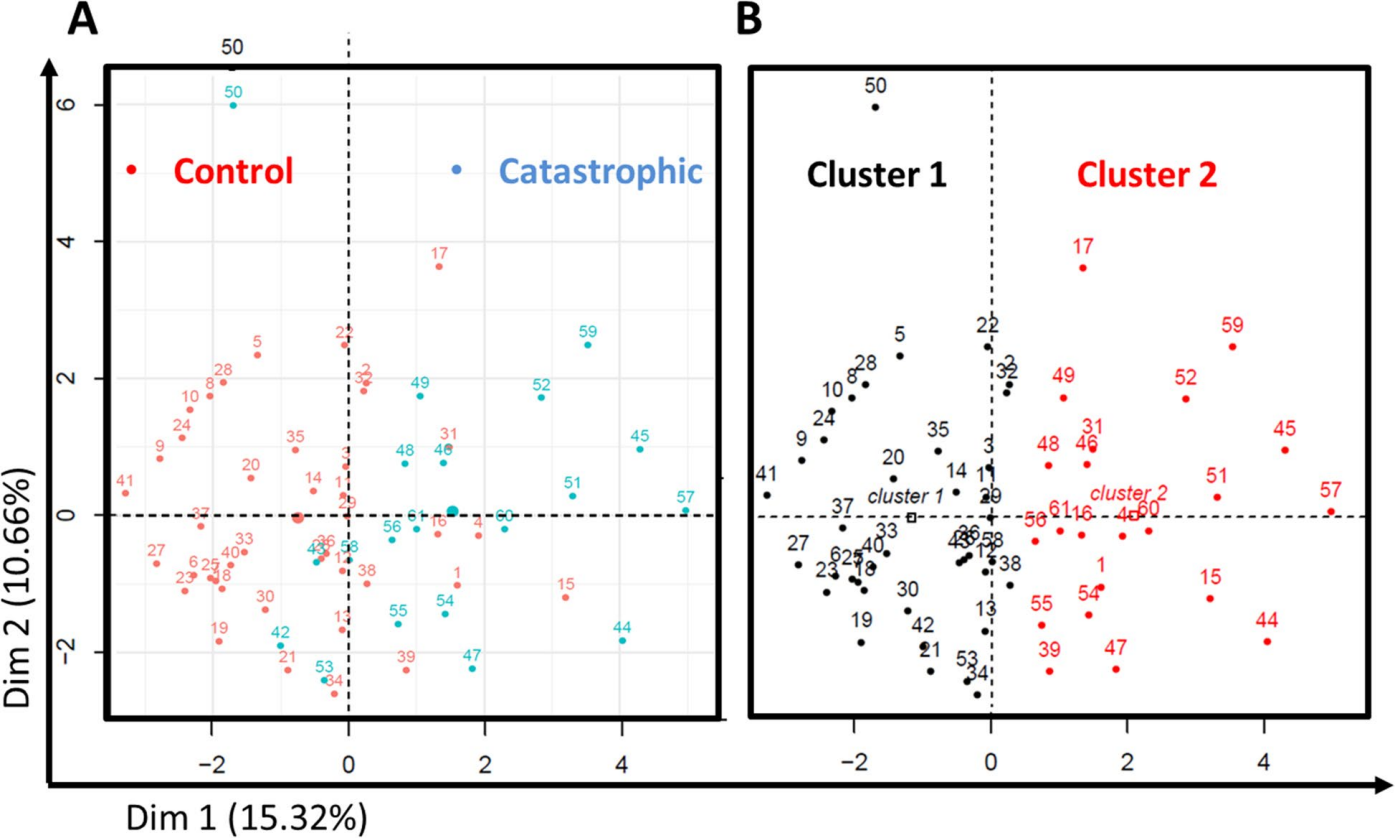
Unsupervised dimension reduction analysis and hierarchical clustering. **A** Factor analysis on mixed data separates 2 groups along axis 1, with strong concordance with patient severity status (catastrophic AOSD, blue; control cases, red; star, mean point of the group). **B** Hierarchical clustering identifies 2 clusters. Cluster 2 (black circles) strongly overlaps with catastrophic AOSD cases

**Fig. 3:**
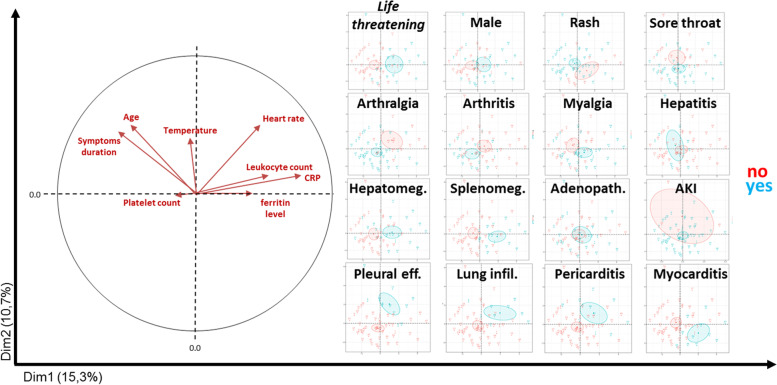
Factor analysis on mixed data (FAMD): axis construction. Contribution of quantitative (left panel) and qualitative (right panel) variables to FAMD axis construction. Adenopath., adenopathy; AKI, acute kidney injury; CAOSD, catastrophic adult-onset Still’s disease; Dim, dimension; Eff., effusion; Hepatomeg., hepatomegaly; infil., infiltrate; Splenomeg., splenomegaly

The first five dimensions of FAMD accounted for 47% of the total inertia of the described/dimension-reduced data frame. The first two axes embodied 15.3% and 10.7% of this inertia, respectively (Fig. 2A). An ascendant hierarchical clustering on FAMD coordinates was performed (Fig. 2B). Two clusters were identified, by visual inspection of inertia gain and by automatic selection. Clustering accuracy to distinguish controls and cases, based on FAMD coordinates, was 80%, with a sensitivity of 75% and a specificity of 83% (*OR* = 13, *95% CI* = 3.4–66, *p* = 2.8E−5). As clearly shown on the first plan of FAMD (Dim1∩Dim2), the two groups of patients were separated, mostly spanning the first axis (Fig. 2A). As detailed in Fig. 3, the variables contributing mostly to the first dimension were C-reactive protein (CRP), blood leukocyte count, splenomegaly, myocarditis and lung infiltrate on one side and age, arthralgia and delay between first symptoms and hospital admission on the other. Overall, these findings are consistent with the hypothesis that even before organ failure CAOSD represents a distinct AOSD subset.

### Young age, early referral and lack of arthralgia as potential red flags for life-threatening complications

In order to determine which key parameters could help identify patients at risk of life-threatening complications, univariate and multivariate statistical analyses were performed to compare CAOSD and uncomplicated cases. CAOSD patients were significantly younger than patients with uncomplicated AOSD and had less arthralgia and arthritis; more frequent hepato-splenomegaly, pulmonary and/or cardiological involvement; and a higher heart rate (Table 2); their CRP and ferritin levels were higher. There were no differences between the two groups in terms of rash, sore throat, serositis, blood cell count or liver enzyme levels. As expected, univariate analyses were in strong agreement with FAMD results.

**Table 2:** Comparison of catastrophic AOSD patients and controls

	Catastrophic AOSD, ***n*** = 20	Control AOSD, ***n*** = 41	Univariate analysis	Multivariate analysis					
				Model 1			Model 2		
			***p***	***p***	Odds ratio	***CI 95%***	***p***	Odds ratio	***CI 95%***
Age	33 (18–64)	40.5 (18–79)	**0.01**	**0.013**	0.94	0.9–0.98			
Male	12 (60%)	18 (44%)	0.24						
Time from first symptoms to hospitalisation	10 (1–61)	20 (1–85)	**0.005**				**0.04**	0.95	0.9–0.99
Fever	19 (95%)	41 (100%)	0.99						
Rash	13 (65%)	35 (85%)	0.07						
Sore throat	13 (65%)	27 (66%)	0.95						
Arthromyalgia	17 (85%)	37 (90%)	0.377						
Arthralgia	10 (50%)	34 (83%)	**0.01**	**0.005**	0.13	0.02–0.49	**0.017**	0.2	0.05–0.93
Arthritis	3 (15%)	19 (46%)	**0.02**						
Myalgia	11 (55%)	21 (52%)	0.78						
Hepatomegaly	11 (55%)	13 (32%)	**0.08**						
Splenomegaly	9 (45%)	8 (19%)	**0.04**						
Adenopathy	10 (50%)	14 (34%)	0.23						
Pleuritis	5 (25%)	7 (17%)	0.47						
Pericarditis	3 (15%)	8 (19%)	0.66						
Lung infiltrate	7 (35%)	2 (5%)	**0.006**						
Myocarditis	8 (40%)	3 (7%)	**0.005**						
Heart rate (/min)	108 (73–150)	95 (70–125)	**0.03**						
Leukocytes (× 10^9^/mL)	16 (1–28)	12 (2–35)	0.11						
Haemoglobin (g/dL)	12 (10–14)	11 (9–14)	0.95						
Platelets (× 10^9^/mL)	262 (142–521)	271 (88–608)	0.47						
Prothrombin time (%)	70 (40–110)	81 (39–123)	0.23						
ASAT (UI/L)	51 (14–615)	51.5 (13–280)	0.25						
ALAT (UI/L)	34 (7–430)	37.5 (6–433)	0.65						
Creatinine (μmol/L)	73 (46–274)	71 (49–314)	0.46						
CRP (mg/L)	272 (61–495)	151 (3–416)	**0.009**						
Ferritin (ng/mL)	27,153 (655–147,568)	6680 (117–49,997)	**0.014**						

*AOSD*, adult-onset Still’s disease; *ASAT*, aspartate aminotransferase; *ALAT*, alanine aminotransferase; *CRP*, C-reactive protein; *CI*, confidence interval; frequency (%); median (min–max)

Variables included in the multivariate analysis: sex, age, age > 50 years, time from first symptoms to hospitalisation, rash, sore throat, arthralgia, arthromyalgia, arthritis, myalgia, hepatomegaly, splenomegaly, adenopathy, pleuritis, lung infiltrate, pericarditis, hepatitis, leukocytes, haemoglobin, platelets, CRP, ferritin and creatinine

Twenty-three variables were included in the multiple logistic regression analysis. Final key variables were arthralgia, age at diagnosis and time from first symptoms to hospitalisation. Due to a significant interaction between age and time from first symptoms to hospitalisation (*ρ* = 0.44, *p* = 6E−4, Pearson), 2 models were built (Table 2), associating arthralgia with either time to hospitalisation (model 1) or age at diagnosis (model 2). Both analyses confirmed that arthralgia was less prevalent in CAOSD than in uncomplicated cases. In the first multivariate model, CAOSD patients were significantly younger than uncomplicated cases. In the second multivariate model, time from first symptoms to hospitalisation was shorter for CAOSD than for uncomplicated AOSD.

## Discussion

AOSD is a rare systemic inflammatory disorder of unknown aetiology [16]. Most studies on the prognosis of AOSD focused on the long-term risk of systemic relapse and/or chronic polyarthritis [5–8]. SJIA and AOSD are currently considered the same diseases at different ages [1]. In the past decade, AOSD has been shown to be a potentially life-threatening disease [9, 10, 17, 18]. As with SJIA, the complication that has attracted most attention is sHLH [9, 10, 19–21]. A variety of organ complications can occur, such as myocarditis, cardiac tamponade, respiratory failure and non-cardiogenic shock [1, 17]. We recently reported a multicentre series of 20 patients that required ICU admission due to AOSD complication-related organ failure, and reviewed 79 published cases [11]. Strikingly, complications mostly occurred at disease onset, resulting in a considerable diagnostic delay, with sepsis as the key misleading working hypothesis.

The main objective of the present study was to determine if CAOSD is a consequence of therapeutic delay or could represent a peculiar disease subset. We found that CAOSD patients tended to have a shorter delay between disease onset and AOSD treatment initiation, which was mainly due to a faster referral to the hospital. Both supervised and unsupervised statistical analyses demonstrated that even before organ failure occurred, these AOSD cases that would subsequently become life-threatening had peculiar characteristics, including a younger age and a shorter interval between disease onset and hospital admission. Furthermore, these CAOSD patients exhibited a peculiar phenotype, with more frequent cardio-pulmonary manifestations, hepato-splenomegaly, higher inflammatory markers and less frequent arthralgia and/or arthritis, when compared to other AOSD patients. Interestingly, these findings are reminiscent of how the heterogeneity of AOSD and SJIA has been delineated in recent years, by opposing arthritis on one side to systemic features and/or macrophage activation on the other [22, 23]. Several pro-inflammatory cytokines play a key role in SJIA/AOSD, including TNF-α, IFN-γ, IL-1, IL-6, IL-17 and IL-18. Ten years ago, Gattorno et al*.* showed that children with SJIA that responded to IL-1 receptor antagonist (anakinra) had less arthritis and higher neutrophilic leucocytosis than others [24]. In 2014, Ichida et al*.* reported that non-arthritic AOSD patients had more frequent splenomegaly, higher ferritin levels and higher IL-18 level and occasionally suffered organ complications (serositis, acute respiratory distress, sHLH, disseminated intravascular coagulation), which were not seen in arthritic patients [25]. More recently, Shimizu et al*.* described 2 cytokine profiles in SJIA: the IL-6-dominant subset had more arthritis, whereas the IL-18-dominant one was more susceptible to sHLH [26]. The same group subsequently made similar observations in patients with AOSD, where IL-18-dominant patients had higher ferritin levels but a lower frequency of arthritis as compared to IL-6-dominant patients [27]. A recent study demonstrated how arthritic versus systemic manifestation of AOSD predicted response to IL-6 and IL-1 blockade, respectively [28].

Overall, our data suggest that CAOSD patients, i.e. AOSD patients who develop life-threatening complications at disease onset, represent a distinct clinical subset characterised by a more rapid and more severe systemic inflammatory response syndrome. This life-threatening cytokine storm might be driven mostly by IL-1 and IL-18. Hyperferritinaemia, hepato-splenomegaly and haemophagocytosis also point to macrophage activation as a key driving force of CAOSD. Our finding that these patients are significantly younger suggests that some genetic susceptibility may play a role.

Importantly, the overwhelming majority of patients who developed life-threatening manifestations did so before the diagnosis of AOSD was made [11]. AOSD is a well-known cause of sHLH, an entity most intensivists are more familiar with. However, key manifestations of most malignancy, immunodeficiency and/or infection-related sHLH include cytopenias. By contrast, despite several features are reminiscent of macrophage activation (fever, hyperferritinaemia, hepato-splenomegaly, coagulopathy and haemophagocytosis), CAOSD present with marked neutrophilic leukocytosis with or without moderate anaemia and/or thrombocytopenia, which likely contribute to misguide clinician [11]. Thus, the main area of improvement is to increase awareness of this peculiar entity among non-specialists (hospitalists, intensivists, respiratory physicians, cardiologists, etc.), who have to be aware of CAOSD being a sepsis mimicker in critically ill adult patients with fever of unknown origin (FUO). Despite the diversity of organ complications that CAOSD patients may present with, the overall picture of AOSD, including the simple clues to its diagnosis, remains the classical ones (salmon rash, sore throat, neutrophil leukocytosis, hyperferritinaemia, etc.). Early recognition of those may avoid pointless investigations, diagnostic delay and deaths of young adult patients.

The exact burden of CAOSD remains unclear in recently published series and discrepancies between them suggest that CAOSD may be underdiagnosed in FUO patients presenting with life-threatening complications. In a large retrospective series from an Italian rheumatological network including 245 AOSD patients, no data were available regarding organ manifestations or deaths, and only 7 patients (2.6%) were considered to have sHLH [2]. In a recent nationwide Japanese series, none of the 169 patients had a fatal outcome [29, 30]. In another French series, patients were seen mainly in internal medicine departments and as many as 33% presented with organ complications (with a heterogeneous spectrum, in line with our own observations [7]). Two recent retrospective AOSD series reported an unexpectedly high mortality rate, which suggests better recognition of CAOSD, but that therapeutic management still needs to be improved. Ahn et al*.* studied 64 Korean patients diagnosed with AOSD at a Seoul Hospital over a 10-year period, of whom 36 (56%) were classified as having sHLH and 12 (18.7%) died [9]. Death occurred within 3 months in all cases (within 1 month in 10 cases), due to AOSD-related multiorgan failure in 8 cases, and superimposed infection in 4. In a recent Italian series of 119 AOSD patients, 19 (16%) patients died due to AOSD: 12 deaths were attributed to AOSD-related sHLH, 5 to AOSD-related MOF and 2 to infection [10]. To date, limited data are available to define the optimum therapeutic strategy beyond corticosteroids. Most published experience reports the promising efficacy of cyclosporin and IL-1 receptor antagonist (anakinra). Intravenous immunoglobulins are an appealing immunomodulatory strategy when infection is a key concern. Unfortunately, they have limited efficacy and may even delay the recourse to more potent therapies [9, 11]. Further studies are needed to clarify the benefit/risk of sHLH-targeting chemotherapy (etoposide) [31], pleiotropic immunosuppressants (e.g. cyclophosphamide) and IL-6 targeted therapies [32] Other candidate drugs will hopefully appear in the near future [32, 33].

Our study has several limitations, owing to its retrospective nature and the rarity of AOSD. Unfortunately, the methodology of case–control collection and the rarity of CAOSD in our single-centre AOSD cohort (5/46, 11%) did not allow us to build a scoring system to predict CAOSD. Thus, the potential red flags we point at should be taken with caution. Another limitation of our study is that we focused on data gathered at first presentation. However, AOSD early clinico-biological course is heterogeneous. Further studies are needed to determine if the dynamics of blood cell counts, liver tests and ferritin levels can predict short-term outcome. Comparing a multicentre case series to a single-centre control group may be a source of bias. However, cases were collected from centres that were very similar to ours (internal medicine department of non-tertiary university or non-teaching hospitals) and 5 were from our own cohort. Further multicentre matched case–controlled studies are warranted to identify red flags for CAOSD.

## Conclusion

There is growing evidence that AOSD can lead to life-threatening complications, mainly before the diagnosis is made. Our study shows that these complications are not the consequence of therapeutic delay but occur in a peculiar subset of patients. It should be popularised that AOSD is a great mimicker in FUO patient entering the ICU. Prospective AOSD cohort studies and interdisciplinary approaches may help to quantify the exact burden of CAOSD. Dedicated studies are needed to identify robust red flags and clarify the optimal therapeutic strategy.

## Data Availability

The datasets analysed during the current study are available from the corresponding author on reasonable request.

## Abbreviations

AOSD: Adult-onset Still’s disease
AKI: Acute kidney injury
ALAT: Alanine aminotransferase
ASAT: Aspartate aminotransferase
BIC: Bayesian criterion information
CAOSD: Catastrophic adult-onset Still’s disease
CI: Confidence interval
CRP: C-reactive protein
FAMD: Factor analysis for mixed data
FUO: Fever of unknown origin
ICU: Intensive care unit
IL: Interleukin
*IQR*: Interquartile range
MOF: Multiorgan failure
NSAID: Non-steroidal anti-inflammatory drug
sHLH: Reactive haemophagocytic syndrome
SJIA: Systemic juvenile idiopathic arthritis
TNF: Tumour necrosis factor
IFN: Interferon
